# Interactions Between Experience, Genotype and Sex in the Development of Individual Coping Strategies

**DOI:** 10.3389/fnbeh.2021.785739

**Published:** 2021-12-20

**Authors:** Rossella Ventura, Simona Cabib, Lucy Babicola, Diego Andolina, Matteo Di Segni, Cristina Orsini

**Affiliations:** ^1^Department of Psychology and Centre for Research in Neurobiology D. Bovet, Sapienza University of Rome, Rome, Italy; ^2^Department of Experimental Neurosciences, IRCCS Fondazione Santa Lucia, Rome, Italy

**Keywords:** dopamine, early stress, gene expression, helplessness, norepinephrine, medial prefrontal cortex, reward, sex differences

## Abstract

Coping strategies, the first line of defense against adversities, develop through experience. There is consistent evidence that both genotype and sex contribute to the development of dysfunctional coping, leading to maladaptive outcomes of adverse experiences or to adaptive coping that fosters rapid recovery even from severe stress. However, how these factors interact to influence the development of individual coping strategies is just starting to be investigated. In the following review, we will consider evidence that experience, sex, and genotype influence the brain circuits and neurobiological processes involved in coping with adversities and discuss recent results pointing to the specific effects of the interaction between early experiences, genotype, and stress in the development of functional and dysfunctional coping styles.

## Introduction

Stress is the main non-genetic source of psychopathology. Therefore, the identification of mechanisms capable of moderating the pathogenic effects of stress is a major goal of clinical and preclinical research. Resilience, the resistance to environmental challenges shown by some individuals, has been the focus of research for the last two decades (Charney, 2004; Southwick et al., 2005; Maier and Watkins, 2010; Daskalakis et al., 2013; Ashokan et al., 2016; Fallon et al., 2020). Indeed, the pathogenic potential of stress experiences does not depend solely on their severity: a large proportion of individuals exposed to traumatic experiences do not develop pathological outcomes (Charney, 2004) and events appraised as positive by most people can be pathogenic stressors for others. Thus, either bereavement or marriage is a potentially pathogenic experience (Paykel, 1997). Humans appraise psychogenic stressors as overwhelming, i.e., demanding beyond their actual means (Folkman et al., 1986; Lazarus, 1993) and this appraisal is accompanied by very high levels of emotional arousal as well as by a stereotypic pattern of physiological responses such as the release of corticotropin-releasing hormone, adrenocorticotropic hormone, and corticosterone/cortisol (Vermetten and Bremner, 2002; Bonne et al., 2004; McEwen and Gianaros, 2011). Both emotional and physiological responses are shared with non-human animals and support the organism’s ability to sustain the challenging experiences. The stress responses have both protective and damaging effects. In the short run, they are essential for adaptation, maintenance of homeostasis, and survival (allostasis). Yet, over longer time intervals, they exact a cost (allostatic load) that can accelerate disease processes (McEwen and Gianaros, 2011). Therefore, once activated, these responses must be terminated as soon as possible. Coping develops from action-oriented and intrapsychic efforts to manage stressors and terminate or moderate stress responses.

When adverse experiences are novel and insensitive to species-typical or previously acquired defensive responses, novel coping strategies are developed through interaction with the stressor, and those appraised as capable of reducing emotional arousal are acquired to be implemented in subsequent encounters with the same or similar stressors (Cabib et al., 2012; Cabib and Puglisi-Allegra, 2012).

Adaptive coping strategies are fitted for the specific characteristic of the stressful experience encountered. However, general categories are recognized in both human and non-human animals. Strategies aimed at eliminating the source of stress, also by escaping/avoiding it, are generally defined as problem-focused or active, whereas those aimed at containing/moderating emotional arousal are defined as passive or emotion-focused. A further distinction is made between reactive and proactive coping strategies: i.e., responses that are directly elicited by the presence of the stressors and those that are elicited by the expectation of a stressful experience (Folkman et al., 1986; Taylor and Stanton, 2007; Coppens et al., 2010; Cabib and Puglisi-Allegra, 2012; Helmreich et al., 2012; Gandhi et al., 2017; Molendijk and de Kloet, 2019; Cabib et al., 2020).

Although the characteristics of dysfunctional coping strategies are still a matter of debate, they have been involved in the development, persistence, and relapse of mental disturbances (Moritz et al., 2016; Sinha et al., 2016; Gandhi et al., 2017; Haskell et al., 2020). Animal models of stress-induced behavioral disturbances are overwhelmingly based on passive coping strategies (Molendijk and de Kloet, 2019; Cabib et al., 2020). Nonetheless, because different coping strategies are effective in different stressful situations, flexible coping is to be considered the healthiest strategy at the individual level (Austenfeld and Stanton, 2004; Coppens et al., 2010; Kent et al., 2017). On the other hand, findings of research in human and non-human subjects support the existence of true coping styles, i.e., a trait-like bias toward the use of a specific coping strategy (Coppens et al., 2010; Fortgang et al., 2016; Santarnecchi et al., 2018; Cabib et al., 2020). Individual coping styles depend on genetic predisposition (Kendler et al., 1991; Koolhaas et al., 1999; Daskalakis et al., 2013; Fortgang et al., 2016) and it has been proposed that individual variance of this phenotype supports population fitness in variable environments, a hypothesis tested by selection studies in mice from wild populations (Koolhaas et al., 1999).

Although determinant, genetic predisposition interacts with individual experience through the lifetime and this interaction can exert a strong influence on individual coping strategies (Moffitt et al., 2006; Daskalakis et al., 2013; Cabib et al., 2019). Moreover, there is increasing evidence for sex-specific adaptation to stress experiences by human and non-human animals (Maeng and Shors, 2013; Kent et al., 2017; Pooley et al., 2018). Finally, certain coping styles, such as proactive coping, are associated with low flexibility and impulsivity in mice (Coppens et al., 2010). These considerations foster the hypothesis that individual coping styles are the results of the interaction between genotype, stress experiences, and sex. A corollary of this hypothesis is that this interaction can foster dysfunctional coping styles leading to psychopathology. As a consequence, the neurobiological mechanisms mediating the development of dysfunctional coping could be strongly involved in psychopathogenesis. The aim of the present review is to discuss evidences from the literature supporting this hypothesis and its corollaries.

This review will discuss results obtained by studies on the effects of experience on the development of coping strategies and on the neurobiological mediators of these effects, focusing on those studies that tested the moderator influence of genotype, sex, or both. To this aim, the review is organized in four sections: a general introduction and four sections each examining findings from studies on experiences known to contribute to the development and stabilization of coping strategies. The studies reported were chosen because they offered data on the effects of genotype, sex, or their interaction.

## Coping with Stress

In humans coping develops from action-oriented and intrapsychic efforts to terminate or moderate emotional and physiological stress responses, thus preventing allostatic load, a most serious health threat (McEwen, 2007). In human research, coping responses are grouped in two broad categories: problem-focused responses target the source of stress and emotion-focused responses directly target emotional arousal (Folkman et al., 1986; Lazarus, 1993). In animal studies, these categories loosely correspond to so-called active and passive coping strategies. The success of coping strategies depends on the stressor. Thus, when stressors are susceptible to action (avoidable/escapable), problem-focused or active coping strategies are most successful. However, when stress is promoted by problems or events devoid of solution, inescapable, or insensitive to the subject’s action (unavoidable/inescapable stressors), the only effective strategies are those aimed at regulating emotional arousal (Austenfeld and Stanton, 2004; Maier and Watkins, 2010; Cabib and Puglisi-Allegra, 2012). Indeed, many reviews of clinical and preclinical data support the adaptive role of passive coping (de Kloet and Molendijk, 2016; Gandhi et al., 2017; Haskell et al., 2020), whereas active avoidance strategies are overexpressed in generalized anxiety disorder, social anxiety disorder, panic, and phobias as well as obsessive-compulsive and post-traumatic stress disorders (Haskell et al., 2020).

Focusing on rodent studies, the Forced Swimming Test (FST), also known as the Porsolt’s test from the name of the researcher who developed it, allows evaluating the progressive adaptation of coping response to a novel uncontrollable/inescapable stress. Indeed, during a first FST experience (10 min for mice 15 min for rats) animals show initial expression of vigorous attempts at escaping from a water tank (reactive coping) by swimming around and struggling to climb the container’s walls. These responses decrease over time whereas episodes of immobility (only small movements required to keep the head above water) increase in frequency and duration. Immobility expressed in FST has been used as a measure of depressive-like behavior; nonetheless, there is increasing consensus on the view that this behavioral response is an adaptive passive coping strategy in FST (Cabib et al., 2012; Andolina et al., 2013; Campus et al., 2015; de Kloet and Molendijk, 2016). Indeed, the immobility response prevents useless and risky loss of energy, thus it is acquired and consolidated as long-term memory to be immediately adopted on subsequent encounters with the stressor (Mitchell and Meaney, 1991; Colelli et al., 2014; Reul, 2014).

**Figure 1:**
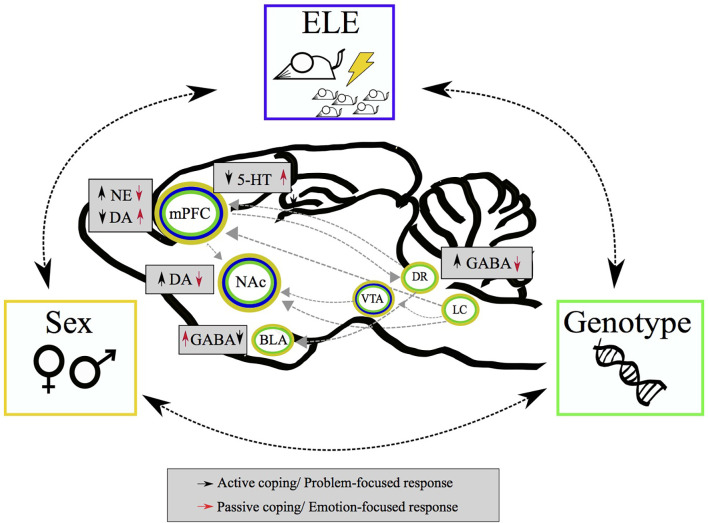
Schematic representation of complex interaction of environmental and individual factors influencing stress coping. Critical experiences in early life, genetic constitutive characteristics, and sex-based differences contribute to coping strategies (i.e., active/passive) mediated by different neurotransmitters balance within mesocorticolimbic circuit and afferent structures.

Experiments performed in rats and mice indicate that enhanced norepinephrine (NE) in the medial pre-frontal cortex (mpFC) supports attempts at active coping in the face of novel stressors by enhancing dopamine (DA) release in the nucleus accumbens shell (NAcSh); whereas a reduction of NE and an increase of DA transmission in mpFC supports the shift toward passive coping in uncontrollable/inescapable stressful conditions by reducing DA availability in the NAcSh (Figure 1). Indeed, mice and rats experiencing a novel uncontrollable/inescapable stressful experience show an immediate increase of DA in the NAc, followed by a decrease below basal or control levels that lasts as long as the experience (Pascucci et al., 2007; Cabib and Puglisi-Allegra, 2012; Latagliata et al., 2014; Di Segni et al., 2016). Manipulations that prevent stress-induced enhancement of NE in mpFC prevent the increase of DA in NAcSh. Instead, manipulations that prevent stress-induced enhancement of DA release in mpFC selectively prevent reduction of DA availability in the NAcSh as well as the development of immobility in the FST (Ventura et al., 2001; Pascucci et al., 2007; Latagliata et al., 2014). The involvement of DA transmission within the corticolimbic system in modulating the expression of passive coping has been subsequently confirmed by optogenetic studies (Chaudhury et al., 2013; Tye et al., 2013; Wenzel et al., 2018; Weele et al., 2019).

A genotype-specific bias toward the expression of passive or active coping responses in the FST has been observed in both rats and mice. Thus, rats selectively bred for rapid acquisition of active avoidance behavior (Roman High Avoidance, RHA) show persistent active coping in the FST in comparison with rats that are slow active avoidance learners (Roman Low Avoidance: RLA; Giorgi et al., 2005, 2007, 2019; Serra et al., 2018). Moreover, male mice genetically selected for short attack latency (SAL) are characterized by prolonged active coping in the FST whereas long-attack latency (LAL) mice show more passive coping in FST and are slower avoidance learners (Koolhaas et al., 1999; Coppens et al., 2010; de Boer et al., 2016). Finally, mice of the DBA/2 (D2) inbred strain show less immobility in the FST than mice of the standard C57BL/6 (B6) strain and outperform B6 mice in protocols involving active avoidance or escape learning (Bovet et al., 1969; Falls et al., 1997; Ventura et al., 2002; Brennan, 2004; Di Segni et al., 2016).

The relationship between good avoidance/escape learning and an active coping style could indicate that neurobiological mechanisms responsible for superior active avoidance learning bias organisms toward the use of active coping strategies. Indeed, acquisition of active defensive responses (i.e., active avoidance, escape) competes with the expression of passive (i.e., freezing, helplessness) defensive strategies (Boeke et al., 2017; Piantadosi et al., 2018; Wenzel et al., 2018). In line with this hypothesis, B6 mice outperform mice of the D2 strain in conditioned freezing (Tipps et al., 2014).

Data on sex differences in coping with FST by rats are conflicting also because in this species behavioral measures are collected on a 5 min test performed 24 h after a first 15 min experience (Drossopoulou et al., 2004). On the other hand, the strain-specific bias toward rapid development of a passive coping response by B6 mice characterizes both female and male mice of this inbred strain when compared with female and male mice of the D2 strains, indicating that this genotype-dependent phenotype is not sex-specific. Moreover, both female and male B6 mice show a rapid increase of mPFC DA and a rapid inhibition of NAc DA when exposed to uncontrollable/unavoidable stressors; whereas female and male D2 mice are characterized by much slower DA responses (Ventura et al., 2002; Di Segni et al., 2016).

Depletion of mpFC DA by local infusion of 6-hydroxydopamine (6OHDA) prevents FST-induced inhibition of mesoaccumbens DA as well as induction of immobility in B6 male mice and a chronic antidepressant treatment moderates FST-induced enhancement of mpFC DA, inhibition of mesoaccumbens DA as well as immobility in male mice of this strain (Ventura et al., 2004). These findings offer strong support to the hypothesis that genotype-dependent variations of mpFC DA response to stress moderate the development of passive coping strategies in inescapable/unavoidable stressful conditions. The shift of mesoaccumbens DA from enhanced to inhibited release during adaptation to a novel inescapable stressor suggests major involvement of a change in the motivational state in the shift from active to passive coping (Cabib and Puglisi-Allegra, 2012). Indeed, NAc DA has been involved in supporting pursuit of highly valued goals despite costs (i.e., time, work, and risk; Salamone et al., 2007; Hauber and Sommer, 2009; Iodice et al., 2017) and in sustaining high rate of response (Niv et al., 2006). Moreover, manipulations that interfere with NAc DA transmission, such as depletion, or local infusion of antagonists, selectively decrease operant response for food while increasing chow intake in rats (Salamone et al., 2007).

Finally, the control exerted by mpFC on mesoaccumbens DA suggests that the shift from high motivational arousal (required to support attempts at active coping) to the motivational blunting (that fosters helplessness) is the outcome of appraisal of the experience as inescapable. Moreover, because mpFC NE transmission is responsible for the increase in the mesoaccumbens DA supporting active coping, this brain area could translate appraisal of a stressful situation into appropriate responses. In line with this view, mpFC can restrain the expression of passive coping and hormonal stress response by activating GABAergic outputs of the anteroventral bed nuclei of the stria terminalis toward the periaqueductal gray and the paraventricular hypothalamic nucleus, respectively (Johnson et al., 2019).

In summary, in a novel stressful situation the development of an active or passive coping strategy is dependent on the impact of the active responses on the stressor appraised by mpFC, and by mesocorticolimbic catecholaminergic transmission.

## Developing Stable Coping Strategies

Individual coping styles can result from the acquisition and consolidation of coping responses through a history of stress (Cabib et al., 2020). Mice and rats with experience of FST show a virtually immediate expression of immobility on subsequent encounters with the stressor and this behavioral response is prevented by manipulations performed immediately following the first FST experience (West, 1990; Colelli et al., 2014; Campus et al., 2015; de Kloet and Molendijk, 2016; Molendijk and de Kloet, 2019). These findings support the view that the immobility response can be an acquired coping strategy that is consolidated as a long-term memory to be expressed on subsequent encounters with the same or similar stressors. Although male mice of both the B6 and D2 strain acquire and consolidate the immobility response in FST, they do so by engaging brain circuits that are only partially overlapping. Indeed, although mice from both strains require a functioning infralimbic cortex (IL) to consolidate memory of the immobility response, B6 mice also require an intact hippocampus whereas D2 mice require a functioning left dorsolateral striatum (Colelli et al., 2014; Campus et al., 2015). This observation further supports the view that coping strategies are acquired and stabilized as long-term memory because the hippocampus is the preferred learning system for mice of the B6 strain but not for mice of the D2 strain (Gerlai, 1998a, b; Ammassari-Teule et al., 2000; Baarendse et al., 2008). Interestingly, in both B6 and D2 mice inactivation of the IL immediately after the extinction of an active coping strategy acquired in a water maze leads to relapse of the extinguished response on the next day, suggesting that acquisition of passive coping in FST involves an extinction-like learning process (Campus et al., 2015).

On the other hand, Learned Helplessness (LH), the generalized but temporary impairment of active coping responses in novel stressful situations, does not seem to involve learning (Maier and Seligman, 2016). LH develops following the experience of stressors that cannot be escaped or controlled. The unique role of the controllability is tested by a protocol involving a rat that experiences trains of shocks at random intervals that can be temporarily interrupted by an operant response: Escapable Stress (ES) and a Yoked one receiving the same amount of shock at the same time being unable to control it, Inescapable Stress (IS; Maier and Seligman, 1976; MacLennan and Maier, 1983; Maier and Watkins, 2005; Baratta et al., 2007; Maier, 2015). IS-exposed rats also develop extinction-resistant conditioned freezing following associative training performed 1 week after stress exposure. Conversely, a previous ES experience potently interferes with subsequent fear conditioning, decreases conditioned freezing expressed in extinction and prevents spontaneous recovery of the extinguished response. Finally, previous ES experience immunizes against the effects of subsequent defeat (Amat et al., 2010; Maier, 2015). Thus, the ability to control a stressful experience determines the development and acquisition of specific coping responses in subsequent encounters with novel stressors.

Controllability does not prevent nor moderate the hypothalamus-pituitary-adrenal (HPA) response to stress (Maier et al., 1986; Prince and Anisman, 1990; Helmreich et al., 2012). Instead, evidence collected by Maier and coworkers indicate that: (1) a corticostriatal circuit connecting mPFC to posterior dorsomedial striatum (DMS) is involved in the appraisal of a stressful experience as controllable/escapable; (2) appraisal of ES inhibits dorsal raphe nucleus (DRN) 5-HT neurons through excitatory inputs from the mpFC to intrinsic GABAergic neurons, leading to a rapid reduction of 5-HT release induced by the shock experience (Maier and Seligman, 2016); and (3) the learned helplessness syndrome is due to desensitization of DRN 5-HT1A inhibitory auto-receptors (Maier, 2015).

Both sex and genotype moderate the behavioral and neural effects of stress controllability. Thus, the proactive effects of control over stress experience are not observable in female rats (Worley et al., 2020). Indeed, although females express the escape response (wheel turning) during ES training as males do, they subsequently show the exaggerated freezing and reduced social exploration that characterize IS-exposed male rats (Baratta et al., 2018, 2019; Fallon et al., 2020). In line with the lack of behavioral effects, ES training does not activate frontocortical neurons responsible for inhibition for the DRN (Baratta et al., 2018) and does not foster circuit-specific plasticity (Baratta et al., 2019) in female rats. Mice of the B6 but not D2 strain develop LH (Shanks and Anisman, 1988). B6 mice are characterized by higher 5-HT1A receptor in PFC and lower GABAb receptors in the basolateral amygdala (BLA) than D2 mice (Popova et al., 1998; Andolina et al., 2014), and when exposed to a novel inescapable/uncontrollable stress they display higher 5-HT outflow in mPFC and higher GABA outflow in the BLA than D2 mice (Andolina et al., 2014).

It should be pointed out, however, that mice of the D2 strain are not resistant to uncontrollable stress but develop a strain-specific dysfunctional adaptation. Indeed, the experience of an uncontrollable/inescapable shock fosters impaired intracranial self-stimulation (anhedonia) and social avoidance in mice of the D2 strain only (Zacharko et al., 1987; Szklarczyk et al., 2012).

In summary, stress coping strategies are strongly influenced by the outcome of previous stress experiences and different mechanisms are responsible for this influence. Thus: (1) learning and extinction learning stabilize active and passive coping strategies, respectively, as responses to similar stressors; (2) previous experience with an uncontrollable stressor fosters a bias toward the expression of passive coping strategies to deal with novel stressors, whereas; and (3) the experience of control over inescapable stress fosters a bias toward the expression of active coping. However, the development of these biases depends on sex- and genotype-specific engagement of cortico-limbic brain circuits and serotoninergic/GABAergic modulation of these circuits (Figure 1).

## Adapting to Environmental Changes

Single severe stressful experiences (acute stressors) reproduce traumas: life and/or health-endangering aversive experiences, in animal models. Traumatic events are the only stressors that can be directly related to a behavioral disturbance (Post-Traumatic Stress Disease: PTSD) in humans. However, a number of far less impressive experiences and even events that most would define as positive and rewarding, known as “life events”, have been associated with the development of physiological and mental disturbances. The severity of these “life events” is measured by stress scales: death of the spouse is most stressful, scoring 100/100 for severity, but marriage scores 50/100 and outstanding achievement 28/100 (Holmes and Rahe, 1967; Monat et al., 1972; Scully et al., 2000). Although life events are discrete experiences, they cause substantial changes and require lasting readjustment of individual’s life; moreover, compelling evidence indicates that a series of moderate life events experienced within a brief period of time (1 or 2 years) are most associated with physical and mental health problems (Monat et al., 1972; Scully et al., 2000). Thus, in non-human subjects, life events are best modeled by chronic or repeated stress experiences.

In restricted feeding protocols, food is removed from the home cage and made available only in specific periods of the day (scheduled feeding). In rodents, this condition reproduces an empty food reserve, thus eliciting the active coping response of foraging. When this protocol is used to motivate learning, it offers foraging-like experiences and, because food is generally made available within the cage after the training session, trained animals experience successful coping. In the absence of training, however, the protocol models a chronic uncontrollable/inescapable stressful experience that fosters compulsive wheel running and enhances behavioral responses to drugs of abuse (behavioral sensitization).

Indeed, food-restricted female rodents can run during the limited hours of food access further reducing feeding, a behavior called activity-based anorexia (ABA). There is evidence that ABA is supported by mesocorticolimbic dysfunctions typically associated with addiction-like neuroplasticity (Broft et al., 2015; Carr, 2020; Milton et al., 2020) and is genotype-specific. Thus, food-restricted female mice of the helplessness-resistant D2 strain but not female mice of the B6 strain develop extreme ABA (Gelegen et al., 2007). It should be pointed out, however, that strong ABA has been reported in adolescent female B6 mice (Wable et al., 2015) suggesting that the strain difference is dependent on developmental processes. In line with this hypothesis, a strain difference in sensitivity to incentive salience of food-associated cues and delayed rewards was only observed in fully adult B6 and D2 mice (Pinkston and Lamb, 2011; Campus et al., 2016; Maiolati et al., 2021).

Rodents exposed to restricted feeding in the absence of a running wheel develop sensitization to the behavioral effects of psychostimulants (Carr, 2002; Sharpe et al., 2012; D’Cunha et al., 2013) a phenomenon fostered by prolonged exposure to addictive drugs and by the experience of IS but not of ES (Maier and Seligman, 1976).

A strain-specific behavioral sensitization to amphetamine is observed in male mice of the D2 strain following either 12 days of restricted feeding or 10 daily experiences of restraint (120 min daily; Badiani et al., 1992; Cabib et al., 2000) suggesting a generalized neuroadaptation to chronic or repeated stress by mice of this inbred strain. In line with this hypothesis, both food-restricted and repeatedly restrained D2 mice show reduced availability of meso-striatal DA receptors of the D2 type (Cabib et al., 1998; Campus et al., 2017), a neuroadaptation fostered by prolonged experience with addictive drugs (Volkow et al., 2007; McCutcheon et al., 2009).

The strain-specific addiction-like neuroplasticity that characterizes food-restricted D2 mice could be driven by corticosteroids. Thus, adrenalectomy only prevents behavioral sensitization fostered by repeated cocaine administration and influences drug-induced neuroplasticity within the mesocorticolimbic DA system in mice of the D2 strain (de Jong et al., 2007, 2008, 2009). Moreover, food-restricted D2 mice show reduced mesocortical and enhanced mesoaccumbens DA in response to restraint stress (Cabib et al., 2002), spontaneous recovery of an extinguished active coping strategy acquired in a water T-maze, and relapse into active coping on retest in the FST protocol (Campus et al., 2015). The latter effect was dependent on reduced availability of dorsal striatal DA receptors of the D2 type (Campus et al., 2017) further supporting the view that aberrant addiction-like neuroplasticity mediates the development of perseverant active coping by mice of the D2 strain. Finally, food-restricted mice of the B6 strain develop perseverant passive coping (Alcaro et al., 2002; Campus et al., 2015), indicating dysfunctional adaptation of genotype-specific coping style.

Chronic and repeated stressful experiences strongly affect gene expression fostered by acute stress challenge in different brain areas. Data obtained comparing mice from the B6 and D2 inbred strains indicate opposite patterns of brain c-fos expression fostered by the first encounter with FST dependent on an interaction between genotype and feeding condition. Indeed, food-restricted male D2 mice were characterized by reduced FST-induced c-fos expression in the IL and in the left dorsolateral striatum and left lateral amygdala; whereas food-restricted male B6 mice showed enhanced FST-induced c-fos expression in the IL, basal and central amygdala, and hippocampus (Campus et al., 2015). Moreover, a genome-wide analysis of basal and stress-induced corticolimbic gene expression performed on brain tissue samples from repeatedly restrained male mice of the two inbred strains challenged with FST (Mozhui et al., 2010) found highly divergent gene expression changes in the mpFC, amygdala, and hippocampus between male B6 and D2 mice with very few genes showing alterations in both strains in any region. Thus, the authors concluded that rather than for the degree of activation of a common molecular “stress network”, mice of the two strains differ for the gene networks engaged by stress.

Slightly different conclusions were reached by a study in female mice of the two inbred strains exposed to chronic mild stress (CMS) protocol and then challenged with a single restraint experience (Terenina et al., 2019). The study reported that nearly all the common transcripts detected in the hippocampus of the stressed mice were increased in B6 and reduced in D2 mice and that mice of the B6 strain were the least responsive to the effects of CMS on the hippocampal transcriptional response to the novel acute stressor. Although the two studies used different stressors, it is tempting to identify a sex-dependent effect of the two genotypes. Indeed, male and female rodents appear to be differentially affected by the chronic/repeated stress procedures, although sex differences depend on the behavioral, physiological, or neurobiological phenotype under screening (Franceschelli et al., 2014).

In summary, adapting to environmental changes can lead to the development of perseverant/inflexible forms of genotype-specific coping styles. Indeed, D2 mice develop perseverant active coping whereas B6 mice develop perseverant passive coping. Moreover, the liability to development of perseverant active coping seems to be age-specific in some genetic background. Finally liability to develop a perseverant active coping style is supported by dysfunctional neuroplasticity within the meso-cortico-limbic system.

## Interaction Between Early Experience, Genotype, and Sex

Early postnatal life represents a particularly relevant time window for individual development, since it is characterized by the extreme sensitivity of neurodevelopment trajectories to environmental influences (Daskalakis et al., 2013; Di Segni et al., 2018; Luby et al., 2020; Nelson and Gabard-Durnam, 2020; Babicola et al., 2021; D’Addario et al., 2021; Lo Iacono et al., 2021). Early experiences can shape synaptic plasticity (Aisa et al., 2009; Lupien et al., 2009; Korosi et al., 2012) permanently affecting brain functioning and modulating behavioral response to stimuli in adulthood (Mintz et al., 2005; Coccurello et al., 2009; Mcclelland et al., 2011). These experiences can regulate flexibility of stress coping strategies adopted in adult life leading to either adaptive or maladaptive coping styles (Pryce et al., 2005; Arborelius and Eklund, 2007; Jezierski et al., 2007; Lupien et al., 2009; Rivarola and Suarez, 2009). Thus, several studies have shown that the same negative events experienced in early life can result in higher susceptibility to mental disturbances but also strengthen resilience to adverse experiences in adulthood (Di Segni et al., 2017, 2018; D’Addario et al., 2021). Finally, precocious aversive experiences can differently impact physical and mental health depending on genetics (Di Segni et al., 2017, 2018).

Early life stress (ELS) is considered a major source of psychopathology and it encompasses different traumatic events experienced during childhood and adolescence (Juruena et al., 2020). One problem with modeling ELS in rodents is maternal adaptation to the stressed pups; indeed, mothers of manipulated pups are, usually, over-caring (Orso et al., 2019). This situation is in sharp contrast with most of the human traumatic conditions that involve absent, reduced or very dysfunctional caregiving and, indeed, can foster long-term positive effects on emotional regulation (D’Amato et al., 1998; Southwick et al., 2005; Daskalakis et al., 2013; Ashokan et al., 2016). However, a recent protocol: repeated cross fostering (RCF), involving a daily change of the fostering mother from postnatal day 1 to postnatal day 4 and leaving the pups with the last adoptive mother until weaning, was shown not to influence maternal behavior, to increase pups’ emotional reactivity to temporary isolation, and to foster hypercapnia in adulthood: an endophenotype of panic disorder in humans (Luchetti et al., 2015).

A complex genotype × sex interaction moderates the long-term effects of RCF on coping with FST. Thus, the early manipulation of the social environment did not influence levels of immobility expressed by adult D2 male mice, it increased levels of immobility expressed by B6 male and D2 females and it decreased immobility levels expressed by B6 females (Di Segni et al., 2016, 2019; D’Addario et al., 2021; Lo Iacono et al., 2021). This variability does not seem to depend on variable sensitivity to the manipulation of the early environment. Indeed, RCF exposure rendered both B6 and D2 pups insensitive to the appeasing effect of nest cues during temporary isolation on postnatal day 8, regardless of the strain; although RCF fostered hypercapnia in B6 mice only (Luchetti et al., 2021). Moreover, in spite of the opposite effects on the behavioral response to FST, RCF did not change baseline or stress-induced corticosterone levels in adult B6 mice, regardless of the sex (Di Segni et al., 2019).

Instead, RCF experience was associated with an enhanced and reduced restraint-induced increase of mpFC DA in D2 and B6 female mice respectively, leading to opposite changes of the mesoaccumbens DA response to the stressor (Di Segni et al., 2016). The strain-specific changes of frontocortical modulation of ventral striatal DA transmission are coherent with the behavioral effect of RCF in mice of each strain (Cabib and Puglisi-Allegra, 2012). Therefore, meso-cortico-limbic DA transmission can be a major target of the long-term effects of early destabilization of the maternal environment. This hypothesis would be in line with the results of studies in human subjects (Gee et al., 2018; Cohodes et al., 2021); moreover, studies of institutionalized children indicate that parental deprivation occurring between 0 and 24 months is especially detrimental for longer-term outcomes (Cohodes et al., 2021). The meso-cortico-limbic system is also involved in modulating motivation toward positive reward and the analysis of saccharine intake by adult RCF-exposed mice revealed an increase of consumption by B6 females and a strong decrease by both B6 male and D2 females (Di Segni et al., 2016, 2019; Lo Iacono et al., 2021), further supporting the impact of RCF on this system.

A complex genotype × sex interaction also moderates the long-term effects of RCF on behavioral and central effects of cocaine in adult mice (Di Segni et al., 2017, 2019; Lo Iacono et al., 2021), in line with previously discussed evidence on the involvement of addiction-like neuroplasticity in the development of dysfunctional active coping.

As discussed in the previous section of this review, stress-induced resistance to develop passive coping toward novel inescapable stressful situations is associated with behavioral sensitization to addictive drugs that depends on the plasticity of the meso-cortico-limbic system. This seems to be the case for the effect of RCF in B6 females. Indeed, RCF-exposed adult female mice of this inbred strain showed enhanced cocaine-induced conditioned place preferences (CPP), NE release in mpFC and DA release in NAc (Di Segni et al., 2017). Opposite effects were observed in RCF-exposed adult B6 mice (Di Segni et al., 2019). Moreover RCF-exposed female D2 mice, showed reduced sensitivity to the effects of cocaine on CPP and on NE release in the mpFC (Di Segni et al., 2017) although DA release fostered by cocaine in the NAc was unaffected by the early experience in these mice because RCF also prevented the cocaine-induced increase of DA in mpFC (Di Segni et al., 2017). Nonetheless, reduced cocaine CPP by RCF-exposed adult mice of the D2 strain could still involve altered functioning of the corticolimbic system. Indeed, the experience of an unstable maternal environment blunted the cocaine-induced c-fos expression in mpFC, NAc, hippocampus, and amygdala of these mice, as well as spinogenesis in mpFC. Opposite effects were observed in adult RCF-exposed female mice of the B6 strain (Di Segni et al., 2017).

It is worth pointing out that phenotypes expressed by RCF-exposed D2 females, i.e., increased helplessness in FST and reduced sensitivity to the behavioral effects of cocaine were reported in adult mice of the D2 strain (sex not specified) raised by adoptive mothers of a different inbred strain (AKR), characterized by low pup-oriented and high nest disturbing behaviors (van der Veen et al., 2008). These effects were strain-specific because they were not observed in adult B6 mice that had experienced the same maternal environment (van der Veen et al., 2008). Because RCF does not affect maternal behavior (Luchetti et al., 2015) the shared long-term effects of the two early manipulations indicate that they are the general outcome of disruption of the mother-infant relationship at the early stages of post-natal development (Lo Iacono et al., 2021), an observation that strongly supports the translational value of the animal models.

Finally, epigenetic modifications (Alyamani and Murgatroyd, 2018) are increasingly recognized as critical to understanding sex differences in brain development and response to early environment (Keller and Roth, 2016). Sex differences in transcriptional signatures of early and adult stress exposure in many brain regions have been reported in both human and animal models (see Brivio et al., 2020 for review; Labonté et al., 2017; Barko et al., 2019; Peña et al., 2019; Parel and Peña, 2020 for review). Data collected in RCF-exposed B6 females indicate a major influence of the early experience on the plasticity of DA neurons located in the ventral tegmental area (VTA; D’Addario et al., 2021), in line with the previously described alteration of the mesocorticolimbic DA response to positive/aversive experiences by these mice (Di Segni et al., 2016, 2017), and very recent findings (Lo Iacono et al., 2021) revealed the role of interaction between sex and genotype on RCF-induced alteration of transcripts in the VTA of adult mice.

In conclusion, the findings obtained by RCF protocol reveal that the development of individual coping styles is moderated by a complex interaction between genotype, sex, and early mother-infant relationship. Moreover, they offer further support to the main role of the meso-cortico-limbic DA system in the expression of coping styles and point to the plasticity of VTA DA neurons as a major mediator of the long-term effects of genotype, sex, and ELS.

## Conclusions

The present review focused on factors influencing coping styles starting from the evidence that dysfunctional coping styles are associated with mental disturbances. The reviewed data support the view that: (1) individual coping styles are genotype-specific; (2) genotype-specific coping styles are moderated by early experience of an unstable mother-pup relationship; (3) individual coping styles can become inflexible/perseverant following chronic/repeated stressful experiences pointing to dysfunctional coping as the outcome of “life events” experienced in the course of the lifetime; (4) aminergic modulation of frontocortical-cortical striatal circuits play a major role in the expression of individual coping styles; and (5) plasticity fostered by a history of stress experiences within these same circuits play a major role in the development of dysfunctional coping.

## Author Contributions

RV and SC planned the review, organized the article, and wrote summary, introduction, and conclusions. DA contributed to section “Developing Stable Coping Strategies”. LB and MD contributed to section “Adapting to Environmental Changes”. CO to section “Coping With Stress”. All authors reviewed and commented the different versions of the article. All authors contributed to the article and approved the submitted version.

## Conflict of Interest

The authors declare that the research was conducted in the absence of any commercial or financial relationships that could be construed as a potential conflict of interest.

## Publisher’s Note

All claims expressed in this article are solely those of the authors and do not necessarily represent those of their affiliated organizations, or those of the publisher, the editors and the reviewers. Any product that may be evaluated in this article, or claim that may be made by its manufacturer, is not guaranteed or endorsed by the publisher.

## Abbreviations

BLA: basolateral Amygdala
DA: dopamine
DR: dorsal Raphe nuclei
ELE: early life experiences
GABA: γ-aminobutyric acid
LC: locus coeruleus
mPFC: medial pre-frontal Cortex
NAc: nucleus accumbens
NE: norepinephrine
VTA: ventral Tegmental Area
5HT: serotonin.
